# The Impact of Bacterial Infections on Delayed Hematopoietic Recovery in Patients with Acute Leukemia After Induction and Consolidation Therapy

**DOI:** 10.3390/jcm15083176

**Published:** 2026-04-21

**Authors:** Krzysztof Gawronski, Nadia Hussein, Agnieszka Woźniak-Kosek, Agata Zakrzewska, Elżbieta Rutkowska, Piotr Rzepecki, Aneta Guzek

**Affiliations:** 1 Department of Hematology and Internal Medicine, Military Medical Institute—National Research Institute, Szaserow Street 128, 04-141 Warsaw, Poland; nhussein@wim.mil.pl (N.H.); przepecki@wim.mil.pl (P.R.); 2 Department of Laboratory Diagnostics, Military Medical Institute—National Research Institute, Szaserow Street 128, 04-141 Warsaw, Poland; awozniak-kosek@wim.mil.pl (A.W.-K.); aguzek@wim.mil.pl (A.G.); 3 Laboratory of Hematology and Flow Cytometry, Department of Hematology and Internal Medicine, Military Medical Institute—National Research Institute, Szaserow Street 128, 04-141 Warsaw, Poland; azakrzewska1@wim.mil.pl (A.Z.); erutkowska@wim.mil.pl (E.R.)

**Keywords:** acute leukemia, acute myeloid leukemia, acute lymphoblastic leukemia, neutropenic fever, bacterial infection, hematopoietic recovery, chemotherapy-induced neutropenia

## Abstract

**Objective:** To evaluate whether documented bacterial infection or neutropenic fever is associated with delayed hematopoietic recovery in patients with acute leukemia undergoing induction or consolidation chemotherapy. **Methods:** We conducted a retrospective observational study of 171 adult patients with acute myeloid leukemia (AML) or acute lymphoblastic leukemia (ALL) treated between 2022 and 2025. Patients were divided into three groups: (1) microbiologically documented infection (*n* = 73); (2) neutropenic fever without pathogen identification (*n* = 73); and (3) no fever or infection (*n* = 25). Hematopoietic recovery was assessed by time to neutrophil recovery (>0.5 × 10^9^/L) and time to reticulocyte production index (RPI) > 1.0. Statistical comparisons were performed using ANOVA or Kruskal–Wallis tests as appropriate. **Results:** The mean time to neutrophil recovery was 32.0 days (95% CI: 30.0–34.0) in Group 1, 28.4 days (95% CI: 27.3–29.6) in Group 2, and 15.2 days (95% CI: 14.3–16.2) in Group 3 (*p* = 0.0039). The mean time to RPI > 1.0 was 36.3 days (95% CI: 34.2–38.4), 33.0 days (95% CI: 31.8–34.2), and 19.4 days (95% CI: 18.5–20.3), respectively (*p* = 0.0018). Differences between Groups 1 and 2 were not statistically significant. **Conclusions:** Infection and neutropenic fever are associated with significantly prolonged hematopoietic recovery following chemotherapy for acute leukemia. Delayed regeneration may increase the risk of complications and negatively affect treatment outcomes.

## 1. Introduction

Acute leukemias are malignant proliferations of immature hematopoietic cells that not only lead to excessive accumulation of blast cells but also severely impair normal hematopoiesis. An important clinical challenge in patients with acute leukemias is the development of infection-associated myelosuppression, which can significantly delay the recovery of leukocytes, erythrocytes, and platelets after chemotherapy. Infections trigger a systemic inflammatory response that further suppresses hematopoietic function and increases the risk of treatment-related complications [1,2].

Emerging evidence indicates that inflammatory mediators directly influence hematopoietic stem and progenitor cells (HSPCs), altering their proliferation, differentiation, and survival. Chronic or severe inflammatory signals, mediated by cytokines such as IL-1, TNF-α, and interferons, can lead to HSPC exhaustion, lineage skewing, and disruption of the bone marrow microenvironment, ultimately contributing to delayed hematopoietic recovery [3,4,5]. These observations suggest that the interplay between infection and stem cell function is a key determinant of hematopoietic restoration following chemotherapy.

Despite growing insights into these mechanisms, there is a notable gap in the literature regarding the quantitative impact of infections on hematopoietic recovery times in patients with acute leukemia undergoing induction or consolidation therapy. Moreover, there is limited information on how different types of infections, including microbiologically confirmed bloodstream infections versus neutropenic fever of unknown origin, influence the kinetics of neutrophil and reticulocyte recovery. Addressing these gaps is critical for optimizing supportive care strategies and chemotherapy scheduling in this vulnerable patient population [6,7,8,9].

At our center, we primarily treat proliferative diseases of the hematopoietic system. Our observations suggest that patients who develop bacterial infections or neutropenic fever tend to experience delayed bone marrow regeneration. In uncomplicated cases, hematopoietic regeneration after chemotherapy usually occurs within approximately 14 days. However, if remission is not achieved after first-line induction therapy, regeneration may be markedly delayed or may not occur at all.

This is because regeneration depends on the return of normal hematopoietic function after therapy. In addition to ineffective induction therapy, infections may also contribute to impaired bone marrow regeneration, a phenomenon demonstrated by Isringhausen et al. in their report. Chronic viral infections are associated with hematopoietic suppression, bone marrow (BM) failure, and hematopoietic stem cell (HSC) exhaustion. However, the ways in which persistent viral challenge and inflammatory responses target BM tissues and perturb hematopoietic competence remain poorly understood [1].

In our previously published work, we demonstrated that fever, which may be a symptom of infection after hematopoietic stem cell transplantation, can cause a delay in hematopoietic regeneration. We showed that the presence of neutropenic fever (NF) resulted in a longer time to engraftment for hematopoietic stem cells [10].

Patients treated for acute myeloid leukemia and acute lymphoblastic leukemia after induction and consolidation chemotherapy always experience pancytopenia, which is a manifestation of bone marrow suppression. Recovery is possible when normal hematopoiesis is restored. However, during the period of pancytopenia, patients remain at high risk of severe infectious complications and death. Not all patients develop an infection, but if an infection occurs, it may delay the regeneration of the hematopoietic system or even lead to the death of the patient.

## 2. Materials and Methods

We conducted an observational, non-randomized retrospective study that included a prospective evaluation of three cohorts of adult patients with acute leukemia (acute myeloid leukemia [AML] and acute lymphoblastic leukemia [ALL]) who developed signs of infection after induction or consolidation chemotherapy.

Patient selection:

Adult patients with acute leukemia treated at the Hematology Clinic of the Military Medical Institute—National Research Institute in Warsaw between 2022 and 2025 were eligible for inclusion.

Inclusion criteria:

- Age ≥ 18 years;
- Diagnosis of acute myeloid leukemia (AML) or acute lymphoblastic leukemia (ALL);
- Treatment with induction or consolidation chemotherapy.

Exclusion criteria:

- Death before hematopoietic recovery could be assessed;
- Incomplete medical records preventing evaluation of hematopoietic recovery.

Group 1 consisted of 73 patients in whom pathogens were isolated from peripheral blood or central venous catheter blood cultures. Bloodstream infections were defined based on positive blood cultures obtained from peripheral veins or central venous catheters.

To distinguish true infections from potential contamination, standard clinical and microbiological criteria were applied, including the presence of clinical signs of infection, repeated isolation of the same pathogen, and consistency with the patient’s clinical course. Cases considered as contamination were excluded from the analysis. These criteria are consistent with widely accepted definitions of bloodstream infections, as described in previous studies [11,12]. The second group (Group 2) consisted of 73 patients with neutropenic fever in whom no pathogen could be identified.

Neutropenic fever was defined as a single oral temperature ≥ 38.3 °C or a sustained temperature ≥ 38.0 °C for at least 1 h in a patient with an absolute neutrophil count < 0.5 × 10^9^/L or expected to decrease below this level [13,14,15].

The third group, Group 3, was a small group of 25 patients with acute leukemia who did not experience any episodes of fever after consolidation therapy. This group was small because, unfortunately, patients most often develop symptoms of neutropenic fever after induction and consolidation therapy, and afebrile patients are a significant minority. The relatively small size of the control group reflects the rarity of patients who do not develop neutropenic fever or infectious complications during intensive chemotherapy for acute leukemia.

The aim of the study was to identify differences in hematopoietic regeneration between patients who underwent the entire course of therapy without any complications and patients who developed infectious complications, especially those in whom a specific pathogen was identified. This study focused on individuals treated at our clinic between 2022 and 2025. Patients were selected from available medical records using predefined inclusion criteria. This means that medical records were randomly selected from three separate groups. This approach was adopted to minimize selection bias, increase the generalizability of the results, improve the representativeness of the sample, and control for potential confounding variables such as age and baseline health status. In addition, we could not recruit patients for the study in advance, as it is unknown at the beginning of treatment whether a patient with acute leukemia will survive the entire treatment phase and whether infectious complications will occur. With this approach to retrospective research based on available medical records, we excluded patients who died during the induction or consolidation phase of treatment due to complications of the disease or treatment process. We did not take these patients into account primarily because they usually did not regenerate their hematopoietic system at all. Blood samples were routinely collected when clinical symptoms suggestive of infection occurred, particularly in the case of fever.

The patients were treated at the Hematology Clinic of the Military Medical Institute in Warsaw.

Study design and ethical approval:

This observational study was conducted in accordance with the Declaration of Helsinki. The study protocol was reviewed and approved by the institutional Bioethical Committee (approval no. 2/26).

All of the factors analyzed and taken into account in our observation were routinely examined in patients treated at our clinic throughout the entire treatment process; therefore, analyzing the data for our observation was easier.

There is not much clear information in the literature on how bone marrow regeneration proceeds in patients with acute leukemia after induction or consolidation who have developed an infection [7,8].

In order to assess bone marrow regeneration, we took into account the increase in neutrophil counts and the corrected reticulocyte count as the most recognizable indicators of regeneration. The ease of such analysis was also related to the fact that patients in our clinic undergoing treatment for acute leukemia have their blood morphology routinely tested on a daily basis. An increase in neutrophil count above 0.5 thousand/μL or an increase in the corrected reticulocyte count above 1.0 was considered the starting point for bone marrow regeneration. It should be noted that our analyzers routinely test absolute and percentage reticulocyte counts automatically, but we calculated the reticulocyte production index ourselves using a mathematical formula.

The Immature Reticulocyte Count (IRC) is calculated by multiplying the percentage of reticulocytes by the percentage of erythrocytes (i.e., hematocrit) and then dividing the result by the patient’s hematocrit value, which is the norm (the accepted norm is 45% or 0.45). The formula is as follows:

IRC = (Percentage of reticulocytes) × (patient’s hematocrit/0.45)

This formula takes into account the impact of anemia on the bone marrow’s ability to produce erythrocytes, which is crucial in assessing whether anemia is regenerative or non-regenerative.

- IRC > 1 suggests a regenerative (productive) response of the bone marrow to anemia.
- IRC < 1 indicates a non-regenerative response of the bone marrow [16].

Among all patients analyzed, the majority had acute myeloid leukemia and a smaller number had acute lymphoblastic leukemia. The detailed characteristics of the patients analyzed are presented in Table 1.

### 2.1. Microbiological Methods

Blood cultures and pathogen detection

Blood samples for microbiological analysis were collected using strict aseptic techniques. For each suspected infectious episode, at least two sets of blood cultures were obtained from separate peripheral venipuncture sites; each set consisted of one aerobic and one anaerobic bottle.

For each bottle, 10 mL of blood was inoculated into BacT/Alert FA Plus (for detection of aerobic bacteria and fungi) and BacT/Alert FN Plus (for detection of anaerobic bacteria) bottles (bioMérieux, 37 Rue de Surène, 75008 Paris, France). Bottles were incubated in the BacT/Alert Virtuo^®^ automated blood culture system (bioMérieux, France) at 37 °C until a positive growth signal was detected or for a maximum of 5 days.

Following a positive signal, a Gram-stained smear was prepared directly from the blood culture bottle. Samples were sub-cultured onto Columbia blood agar, chocolate agar, MacConkey agar, and Sabouraud agar. Material from anaerobic blood culture bottles was additionally inoculated onto Schaedler agar. Aerobic plates were incubated at 37 °C in ambient air supplemented with 5% CO_2_, while Schaedler agar plates were incubated under anaerobic conditions for 48 h.

Microorganisms were identified using matrix-assisted laser desorption/ionization time-of-flight mass spectrometry (MALDI-TOF MS) with the VITEK^®^ MS system (bioMérieux, France).

In cases where multiple blood cultures were positive during a single clinical episode, only the first isolate per episode was included in the analysis.

### 2.2. Antimicrobial Susceptibility Testing

Antimicrobial susceptibility testing (AST) was performed using the VITEK^®^ 2 automated system (bioMérieux, France) in accordance with the manufacturer’s instructions. For Gram-negative bacteria, AST-N331 and AST-N332 cards were used, while AST-P644 and AST-P643 cards were applied for Gram-positive organisms.

Susceptibility results were interpreted according to the European Committee on Antimicrobial Susceptibility Testing (EUCAST) guidelines valid at the time of the study. Colistin susceptibility was determined separately using the reference broth microdilution method with the Micronaut MIC-Strip colistin (MMS) assay (Merlin Diagnostika GmbH, Kleinstr 14, 53332 Bornheim, Germany).

Quality control procedures were performed using the following reference strains: *Pseudomonas aeruginosa* ATCC 27853, *Escherichia coli* ATCC 25922, *Staphylococcus aureus* ATCC 29213, *Enterococcus faecalis* ATCC 29212, *Klebsiella pneumoniae* ATCC 700603, *Klebsiella pneumoniae* ATCC BAA-2814, *Staphylococcus aureus* NCTC 12493, and *Enterococcus faecalis* ATCC 51299. When clinically significant resistance mechanisms were suspected, additional confirmatory tests were performed.

### 2.3. Detection of Antimicrobial Resistance Mechanisms

Phenotypic detection of extended-spectrum β-lactamase (ESBL) production was carried out using the double-disk synergy test (DDST), as described previously [17].

The presence of carbapenemase-producing organisms was assessed using a lateral flow immunochromatographic assay (RESIST-5 O.O.K.N.V., Coris BioConcept, Gembloux, Belgium), enabling detection of the most common carbapenemase families.

Methicillin resistance in Staphylococcus aureus was determined using the cefoxitin disk diffusion method. A zone diameter of <20 mm was interpreted as resistant, in accordance with established criteria [18].

Vancomycin resistance in enterococci was assessed using the vancomycin E-test (bioMérieux, France), performed according to the manufacturer’s instructions. Isolates with minimum inhibitory concentration (MIC) values ≥ 32 µg/mL were classified as vancomycin-resistant enterococci (VRE).

### 2.4. Statistical Methodology

Statistical analyses were performed using Statistica 13.3 (TIBCO Software Inc., Palo Alto, CA, USA) at https://statistica.software.informer.com/13.3/ (accessed on 12 May 2025).

Quantitative variables are characterized using the arithmetic mean, standard deviation, median, minimum and maximum values (range), and 95% confidence intervals (CIs).

Qualitative variables are presented using counts and percentages (percentage).

The Shapiro–Wilk test was used to check whether the quantitative variable indicated a normally distributed population. The Levene (Brown–Forsythe) test was used to test the hypothesis of equal variances.

The significance of the differences between the two groups was examined using significance tests: Student’s *t*-test or the Mann–Whitney U test. The significance of differences between more than two groups was checked using the F test (ANOVA) or the Kruskal–Wallis test. In the case of there being statistically significant differences between the groups, post hoc tests were used (Tukey’s test for F, Dunn’s test, or the Kruskal–Wallis test).

Correlation analysis was used to calculate Pearson’s and/or Spearman’s correlation coefficients and determine the relationship, strength, and direction between the variables.

In all calculations, a significance level of *p* = 0.05 was established. For graphical comparison of recovery-time distributions between study groups, kernel density plots were used.

## 3. Results

Baseline characteristics of the study groups were comparable. No statistically significant differences were observed with respect to age, sex distribution, leukemia type (AML vs. ALL), or treatment phase (induction vs. consolidation).

Various types of chemotherapy were used in the treatment of patients; all of these treatments are presented in Table 2.

The main aim of our study was to assess the impact of bacterial infections on the time to hematopoietic recovery in patients with acute leukemia undergoing induction and consolidation therapy. Therefore, the analysis specifically focused on patients in whom hematopoietic recovery could be evaluated.

A total of 14 patients were excluded from the analysis due to early mortality related to infections: six patients died after induction therapy and three patients died after consolidation therapy due to bacterial infections. In addition, four patients died due to neutropenic fever after induction therapy, and one patient died due to neutropenic fever after consolidation therapy. These patients were excluded because hematopoietic recovery could not be assessed in their cases.

We recognize that severe infections can lead to early mortality, preventing the occurrence—and thus assessment—of hematopoietic recovery. In such cases, it is impossible to estimate the impact of infection on delayed regeneration because the endpoint of interest cannot be observed. Therefore, patients who died before hematopoietic recovery were excluded from the analysis.

### Legend

Induction therapy refers to the initial intensive chemotherapy aimed at achieving complete remission, while consolidation therapy refers to subsequent chemotherapy cycles intended to eradicate residual disease and maintain remission. These definitions are clearly indicated to ensure proper understanding of the treatment phases. Patients who did not achieve remission after first-line induction therapy were treated with a second induction regimen of different composition.

Among the 73 individuals in Group 1 in whom a specific bacterium was identified, there were 29 women and 44 men. Bacteremia was identified in 30 cases from peripheral veins and in 43 cases from blood collected from central catheters. Table 3 presents a list of isolated pathogens.

**Figure 1:**
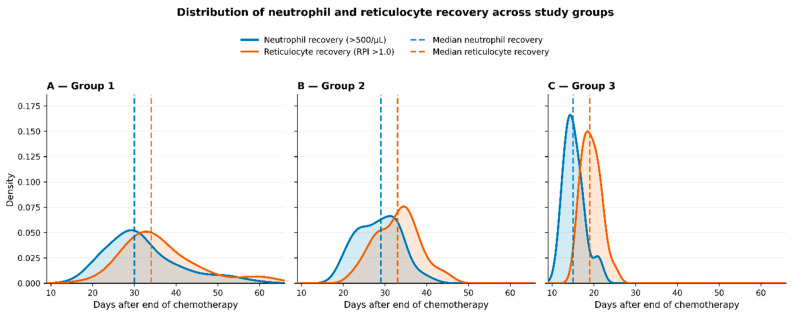
Distribution of neutrophil and reticulocyte recovery across study groups.

The analysis of hematopoietic recovery demonstrated significant differences between the study groups (Table 4, Figure 1).

Patients with microbiologically documented infection (Group 1) exhibited the longest time to hematopoietic recovery. The mean time to neutrophil recovery (>0.5 × 10^9^/L) was 32.0 days (95% CI: 30.0–34.0), while the mean time to reticulocyte recovery (RPI > 1.0) was 36.3 days (95% CI: 34.2–38.4).

In patients with neutropenic fever without microbiological confirmation (Group 2), recovery times were slightly shorter but remained prolonged. The mean time to neutrophil recovery was 28.4 days (95% CI: 27.3–29.6), and the mean time to reticulocyte recovery was 33.0 days (95% CI: 31.8–34.2).

In contrast, patients without fever or documented infection (Group 3) showed substantially earlier hematopoietic recovery. The mean time to neutrophil recovery was 15.2 days (95% CI: 14.3–16.2), and the mean time to reticulocyte recovery was 19.4 days (95% CI: 18.5–20.3).

Statistically significant differences were observed between the three groups for both neutrophil recovery (*p* = 0.0039) and reticulocyte recovery (*p* = 0.0018). However, the differences between Group 1 and Group 2 were not statistically significant.

These differences are further illustrated in Figure 1, which presents the distribution of recovery times using kernel density plots. In all groups, neutrophil recovery preceded reticulocyte recovery, reflecting earlier restoration of the myeloid lineage. Group 1 demonstrated the most delayed and heterogeneous erythroid recovery, with the peak distribution occurring approximately 30–35 days after chemotherapy. Group 2 showed an intermediate pattern, with a slightly earlier and less dispersed distribution. In contrast, Group 3 exhibited earlier and more homogeneous recovery of both neutrophils and reticulocytes, with a markedly narrower distribution.

Overall, these findings indicate that infectious complications are associated not only with prolonged hematopoietic recovery but also with increased variability in the timing of regeneration.

Panels A–C present kernel density estimates of time to neutrophil recovery (>0.5 × 10^9^/L) and time to reticulocyte production index (RPI) > 1.0 in Groups 1–3, respectively. Dashed vertical lines indicate median recovery times for each parameter. In all groups, neutrophil recovery preceded reticulocyte recovery, consistent with earlier restoration of the myeloid lineage compared to the erythroid lineage. However, marked differences were observed in the timing and dispersion of recovery between groups. Group 1 showed the most delayed erythroid recovery, with the highest density of reticulocyte recovery occurring approximately 30–35 days after chemotherapy. Group 2 demonstrated an intermediate pattern, with delayed but less dispersed recovery than Group 1. In contrast, Group 3, comprising patients without fever or documented infection, showed earlier and more synchronized recovery of both lineages, with a narrower distribution of recovery times. These findings illustrate that infectious complications are associated not only with longer recovery times, but also with greater variability in hematopoietic regeneration.

## 4. Discussion

Chemotherapy remains a cornerstone of treatment for acute leukemia; however, it is associated with significant toxicity to normal hematopoietic cells, resulting in neutropenia, anemia, and thrombocytopenia. Hematopoietic stem cells (HSCs) play a central role in post-chemotherapy recovery, although the mechanisms regulating their activation and regeneration remain incompletely understood.

In this study, we demonstrate that infectious complications are associated with both delayed and more heterogeneous hematopoietic recovery following chemotherapy. This effect was consistently observed in both quantitative analyses and graphical visualization (Figure 1).

The use of kernel density plots provided additional insight into recovery dynamics beyond mean values. Patients with microbiologically documented infection (Group 1) exhibited not only prolonged recovery times but also broader distributions of reticulocyte recovery, indicating increased variability in erythroid regeneration. Patients with neutropenic fever (Group 2) demonstrated a similar but less pronounced pattern, suggesting that systemic inflammation alone may contribute to delayed recovery. In contrast, patients without fever or infection (Group 3) showed earlier and more homogeneous regeneration, indicating a more predictable recovery process in the absence of inflammatory stress.

Importantly, our findings suggest that infection influences not only the timing but also the variability of hematopoietic recovery. Increased variability may reflect instability in bone marrow regeneration and may complicate clinical decision-making, including prediction of recovery, timing of subsequent chemotherapy cycles, and the need for supportive care.

These observations can be explained by infection-driven alterations in hematopoietic stem and progenitor cell (HSPC) function and the bone marrow microenvironment. Pro-inflammatory cytokines such as IL-1, TNF-α, and interferons are known to modulate HSPC proliferation and differentiation [2,3,4,5,6]. While acute inflammatory signaling may transiently enhance myelopoiesis, sustained inflammation can lead to HSPC exhaustion, impaired self-renewal, and lineage skewing toward myelopoiesis at the expense of erythropoiesis [1,2,3,4,5,6].

Delayed hematopoietic recovery may increase supportive care requirements and prolong hospitalization, as suggested by previous studies in patients with hematologic malignancies [19,20,21,22,23,24]. Although our study was not designed to assess mortality, transfusion burden, or treatment delays directly, our findings suggest that infection-related impairment of hematopoiesis may have clinically relevant consequences.

In addition, infection-associated inflammation may disrupt the bone marrow niche, including mesenchymal stromal cells and other regulatory components essential for hematopoietic homeostasis [1,5,25,26,27]. Damage to this microenvironment may impair coordinated regeneration, contributing to the increased variability observed in Figure 1.

Additional indirect mechanisms may also play a role. Antibiotic therapy, immune-mediated processes, and alterations in the gut microbiome have all been implicated in the modulation of hematopoiesis [28,29,30,31,32], potentially further contributing to delayed and heterogeneous recovery.

Erythropoiesis is particularly sensitive to inflammatory stress, with cytokine-mediated suppression of erythroid progenitors and inflammation-related disturbances in iron metabolism further contributing to delayed red cell recovery [33].

Our findings are also consistent with previous observations suggesting that febrile or inflammatory complications may delay hematopoietic recovery. In particular, Gawronski, Tomasiuk, Wcislo et al. demonstrated that inflammatory complications after hematopoietic stem cell transplantation were associated with delayed engraftment and altered recovery dynamics [10]. Although the clinical setting in their research differs from that of the present study, that report supports the concept that inflammatory stress may adversely affect hematopoietic regeneration.

This study has several limitations. The heterogeneity of chemotherapy regimens reflects real-world clinical practice but may have influenced recovery dynamics. The retrospective design may introduce bias, and the exclusion of patients who died before recovery may result in survivorship bias. In addition, the relatively small size of the control group reflects the clinical reality that most patients undergoing intensive chemotherapy develop infectious complications. Despite these limitations, the observed differences were statistically significant and consistent across analyses.

Taken together, our findings support the concept that infection-related inflammatory processes not only delay hematopoietic recovery but also disrupt its regulation, leading to less predictable regeneration dynamics. Although the present study is primarily descriptive, it provides quantitative real-world evidence supporting the clinically relevant association between infectious complications and delayed hematopoietic recovery. These findings may serve as a foundation for future studies incorporating predictive modeling and risk stratification approaches.

## 5. Limitations

This study has several limitations that should be acknowledged. First, the retrospective design may introduce selection bias and limits the ability to establish causal relationships between infectious complications and delayed hematopoietic recovery. Second, the control group of afebrile patients was relatively small compared with the other groups, reflecting the fact that most patients undergoing intensive chemotherapy for acute leukemia develop neutropenic fever or infectious complications. Third, patients who died before hematopoietic recovery could be assessed were excluded from the analysis. This may introduce survivorship bias, as the most severe infections could have resulted in early mortality before recovery occurred.

In addition, patients received heterogeneous chemotherapy regimens during induction and consolidation therapy, which may have influenced the dynamics of hematopoietic recovery. Finally, we did not perform multivariable analyses due to sample size limitations, which may have reduced the ability to adjust for potential confounders such as leukemia subtype or treatment phase. Future prospective studies including larger patient cohorts and multivariable approaches are warranted to further clarify the relationship between infection and delayed hematopoietic regeneration.

## 6. Conclusions

Our study demonstrates that infectious complications and neutropenic fever are associated with significantly delayed hematopoietic recovery after induction or consolidation chemotherapy in patients with acute leukemia. Prolonged bone marrow regeneration may increase the risk of treatment complications and adversely affect clinical outcomes. Further prospective studies are needed to better understand the mechanisms linking infection and impaired hematopoiesis.

## Figures and Tables

**Table 1:** Characteristics of the patients that qualified for the groups.

Cohort	Group 1 (*n* = 73)	Group 2 (*n* = 73)	Group 3 (*n* = 25)
Sex			
Female	29 (39.7%)	33 (45.2%)	10 (40.0%)
Male	44 (60.3%)	40 (54.8%)	15 (60.0%)
Age	50.5 ± SD 7.9	51.3 ± SD 6.9	49.0 ± SD 6.0
Induction Remission Chemotherapy	36 (49.3%)	40 (54.8%)	13 (52%)
Consolidation Remission Chemotherapy	37 (50.7%)	33 (45.2%)	12 (48%)
Diagnosis			
AML	61 (83.6%)	64 (87.7%)	22 (88%)
ALL-B	12 (16.4%)	9 (12.3%)	3 (12%)

**Table 2:** Types of chemotherapy used for the patients included in the study.

Disease	Types of Chemotherapy
AML	induction DA 3 + 7 Ara-C + daunorubicin
	induction DA 3 + 7 HD Ara-C + daunorubicin + gemtuzumab ozogamicin
	induction DA 3 + 7 HD Ara-C + daunorubicin + midostaurin
	consolidation high dose (HD)-AraC
	consolidation HD ARA-C + gemtuzumab ozogamicin
	consolidation HD ARA-C + midostaurin
	consolidation HAM—high doses (HD) ARA-C + mitoxantrone
	consolidation HD ARA-C + gemtuzumab ozogamycin PALG > 60 y.o.
	second induction CLAG-M (cladribine, AraC, Mitoxantron, G-CSF)
	second induction Nove-HiDAC
ALL	induction PALG (Polish adult leukemia group) ALL-B < 55 rż (Vincristine + Daunorubicyne + PegAsparaginase)
	induction PALG ALL7 Ph(^−^) < 55 rż, (vincristine + dauonorubicin + rituximab + pegaspargase)
	consolidation PALG ALL7Ph > 55 rż OUN + (HD methotrexate + etoposid)

**Table 3:** Characteristics of isolated bacteria.

Pathogen	Number of Isolates	Percentages (%)
*Staphylococcus epidermidis*	17	23.3
*Klebsiella pneumoniae*	14	19.2
*Enterococcus faecium*	11	15.1
*Enterococcus faecalis*	3	4.1
*Stenotrophomonas maltophilia*	3	4.1
*Listeria monocytogenes*	1	1.4
*Escherichia coli*	7	9.6
*Staphylococcus aureus*	7	9.6
*Streptococcus mitis*/*Streptococcus oralis*	1	1.4
*Klebsiella oxytoca*	1	1.4
*Morganella morganii*	1	1.4
*Candida glabrata*	2	2.7
*Bacillus cereus*	1	1.4
*Streptococcus pneumoniae*	1	1.4
*Candida krusei*	1	1.4
*Pseudomonas aeruginosa*	2	2.7

**Table 4:** Parameters of hematopoietic recovery.

Parameters of Hematopoietic System Regeneration	Patients with Identified Infection After Chemotherapy Group 1 (*N* = 73)	Patients with Neutropenic Fever Group 2 (*N* = 73)	Patients Without Infection and Without Fever Group 3 (*N* = 25)	*p* < 0.05 1–2–3 ANOVA Kruskal–Wallis
Reticulocyte recovery (days, RPI > 1.0)	36.3 (95% CI: 34.2–38.4)	33.0 (95% CI: 31.8–34.2)	19.4 (95% CI: 18.5–20.3)	0.0018
Neutrophil recovery (days)	32.0 (95% CI: 30.0–34.0)	28.4 (95% CI: 27.3–29.6)	15.2 (95% CI: 14.3–16.2)	0.0039

## Data Availability

The data supporting the findings of this study are available from the corresponding author upon reasonable request.
